# Regulating cancer risk prediction: legal considerations and stakeholder perspectives on the Canadian context

**DOI:** 10.1007/s00439-023-02576-8

**Published:** 2023-06-26

**Authors:** Palmira Granados Moreno, Terese Knoppers, Ma’n H. Zawati, Michael Lang, Bartha M. Knoppers, Michael Wolfson, Hermann Nabi, Michel Dorval, Jacques Simard, Yann Joly

**Affiliations:** 1 Centre of Genomics and Policy, Faculty of Medicine, McGill University, Montréal, Québec Canada; 2 School of Epidemiology and Public Health, University of Ottawa, Ottawa, Ontario Canada; 3 Oncology Division, CHU de Québec-Université Laval Research Center, Québec City, Québec Canada; 4 Department of Social and Preventive Medicine, Faculty of Medicine, Université Laval, Québec City, Québec Canada; 5 Faculty of Pharmacy, Université Laval, Québec City, Québec Canada; 6 CISSS Chaudière-Appalaches Research Centre, Lévis, Québec Canada; 7 Genomics Center, CHU de Québec-Université Laval Research Center, Québec City, Québec Canada; 8 Department of Molecular Medicine, Université Laval, Québec City, Québec Canada

## Abstract

Risk prediction models hold great promise to reduce the impact of cancer in society through advanced warning of risk and improved preventative modalities. These models are evolving and becoming more complex, increasingly integrating genetic screening data and polygenic risk scores as well as calculating risk for multiple types of a disease. However, unclear regulatory compliance requirements applicable to these models raise significant legal uncertainty and new questions about the regulation of medical devices. This paper aims to address these novel regulatory questions by presenting an initial assessment of the legal status likely applicable to risk prediction models in Canada, using the CanRisk tool for breast and ovarian cancer as an exemplar. Legal analysis is supplemented with qualitative perspectives from expert stakeholders regarding the accessibility and compliance challenges of the Canadian regulatory framework. While the paper focuses on the Canadian context, it also refers to European and U.S. regulations in this domain to contrast them. Legal analysis and stakeholder perspectives highlight the need to clarify and update the Canadian regulatory framework for Software as a Medical Device as it applies to risk prediction models. Findings demonstrate how normative guidance perceived as convoluted, contradictory or overly burdensome can discourage innovation, compliance, and ultimately, implementation. This contribution aims to initiate discussion about a more optimal legal framework for risk prediction models as they continue to evolve and are increasingly integrated into landscape for public health.

## Introduction

Risk prediction has gained traction in recent years for its potential in clinical oncology (Drabiak 2017; Wand et al. 2021). Models that assess how a patient might respond to treatment, or that can forecast whether an individual is likely to develop cancer in the first place, stand to assist both physicians and patients in making more reliable and informed therapeutic or preventive decisions (Usher-Smith et al. 2015; Lee et al. 2019). Cancer is the leading cause of death in Canada, and is frequently diagnosed at advanced stages where treatment is least effective and prognosis is most poor (Wang et al. 2014; Brenner et al. 2022). Risk prediction models could support the development of more accurate and effective guidelines for screening modalities, frequencies and ages (Lee et al. 2019; Knoppers et al. 2021). They could also improve the counseling process, providing clinicians and patients with risk-adapted clinical pathways and the additional options that come with advanced warning of cancer risk. Ultimately, the targeted screening and early prevention, detection and intervention mechanisms associated with susceptive risk prediction models hold potential for reducing the impact of cancer in society via prolonged and saved lives (Lee et al. 2014, 2019; Usher-Smith et al. 2015; Knoppers et al. 2021).

Though cancer risk prediction holds great promise (Usher-Smith et al. 2015), questions about the regulatory compliance requirements applicable to these models raise legal uncertainty. It is not obvious, for example, whether risk prediction tools are subject to regulatory requirements for pre-market review, manufacturer licensing, and post-market safety assessment. One possibility is that they will be regulated according to the framework usually applicable to software as medical devices (SaMD). Because risk prediction models are principally aimed at *predicting* the incidence of disease, often well in advance of clinical presentation and symptom onset, they could be seen to perform a specific and potentially unique medical purpose. Medical devices are typically understood to be technologies engaged in diagnosing, treating, mitigating, or preventing disease. Though risk prediction could theoretically assist with each of these functions, it generally only does so indirectly. Susceptive risk prediction appears to perform a set of medical functions that may not have been fully envisioned by existing regulation. By the same measure, if risk prediction models are to be regulated, they would likely be regulated under emerging regimes for SaMD. However, these novel SaMD oversight frameworks raise their own unanswered questions, especially surrounding their exclusion criteria. All of this means that risk prediction models might be in a poorly defined legal space, with significant uncertainty for stakeholders about whether and how these new tools are regulated.

This paper examines the regulatory compliance requirement for risk prediction models in the context of Canadian medical devices regulation highlighting in the process how the Canadian approach compares to what currently applies in the U.S. and Europe. It consists of three parts. In Part One, we broadly describe how medical devices, including SaMD, are regulated in Canada. In Part Two, we more precisely define susceptive cancer risk prediction models and outline examples. We then apply the legal framework described in Part One to risk prediction models. We use as an exemplar the BOADICEA risk prediction model that computes an individual’s prospects of carrying rare loss-of-function variants in certain breast and ovarian cancer susceptibility genes and their relative risks of developing breast or ovarian cancer (Lee et al. 2019). In Part Three, we present general findings from a series of qualitative interviews we held with expert stakeholders from four key groups: researchers and developers, regulators and decision-makers, patient advocates and the medical technology industry. This section provides a glimpse at the accessibility and compliance challenges of the Canadian SaMD framework as it applies to risk prediction models. It also identifies areas that may need clarification, and potential problems for policymakers to address. We conclude by noting that both cancer prediction and SaMD regulation are rapidly evolving and that staying apprised of developments in both areas will be essential for improving predictive modeling and associated health outcomes.

## Medical devices regulation

Part One gives a short overview of the way medical devices are regulated in Canada. Two sources of law structure much of the sale and distribution of medical devices: *the Food and Drugs Act (Act)* (Food and Drugs Act 1985) and the *Medical Devices Regulations* (*Regulations*) (Medical Devices Regulations 1998). Both the *Regulations* and medical devices provisions of the *Act* are administered by the Medical Devices Directorate (MDD), a division of Health Canada broadly charged with regulating, monitoring, and evaluating medical devices (Health Canada 2021). The MDD works to ensure that medical devices are safe and perform as expected (Health Canada 2021). These goals are advanced through a series of pre-market and post-market controls, including certain conditions for labeling and packaging, stipulations with respect to sale and advertising, and periodic safety surveillance obligations (Thorogood et al. 2018; Health Canada 2021). The level of regulatory oversight to which a medical device is subject depends on the extent of potential risk the device is deemed to pose to patients. Thus, riskier devices generally are subject to stricter regulatory requirements.

To address the shifting nature of medical devices development, Health Canada created the Digital Health Review Division (DHRD) of the MDD in 2018 to specifically oversee digital medical devices, including wireless medical devices, mobile medical apps, telemedicine, and artificial intelligence (Health Canada 2018a). Also included in the DHRD’s mandate is organizing the review of SaMD according to the *Guidance Document for Software as a Medical Device: Definition and Classification* (SaMD Guidance) (Health Canada 2018a, 2019a, b). We describe the scope of the SaMD Guidance Document in more details below.

### Key definitions

The regulation of medical devices is a complex assembly of rules, exceptions, and principles. To understand how the regulatory regime operates, it is necessary to first consider how its basic concepts are defined. For our purposes, three key terms warrant special attention: medical devices, medical purpose, and SaMD.

#### Medical devices

The *Regulations* define ‘medical device’ as an “instrument, apparatus, contrivance or other similar article, or an in vitro reagent, including a component, part or accessory of any of them, that is manufactured, sold or represented for use in diagnosing, treating, mitigating or preventing a disease, disorder or abnormal physical state, or any of their symptoms in human beings” (Medical Devices Regulations 1998). According to this definition, a medical device can include a wide range of tools and on its face appears to include risk prediction models in its scope (Thorogood et al. 2018). The *Regulations* further distinguish *active* from *inactive* devices, the former of which refers to devices powered by a source of energy other than energy generated by the human body or gravity (Medical Devices Regulations 1998). In vitro devices (IVDD) are further outlined as medical devices intended to be used in vitro for the examination of specimens taken from the body (Medical Devices Regulations 1998).

#### Medical purpose

The concept of a medical purpose plays a dominant role in determining whether a software is likely to be classified and regulated as a medical device. In the SaMD context, Health Canada specifies that “when the intended or represented use of software is for one or more of the medical purposes set out in the definition of a device as stated in the [*Food & Drugs Act*], that software qualifies as a medical device” (Health Canada 2019a). Relevant medical purposes might include diagnosing, treating, preventing, or restoring a disease, disorder, or abnormal state, or any of its symptoms. In the context of cancer risk prediction, diagnosis may be an especially salient medical purpose. It is generally understood to consist of examining “specimens for the purpose of providing information concerning a physiological state, state of health or disease or congenital abnormality” (Health Canada 2019a).

#### SaMD

Health Canada’s SaMD Guidance Document defines SaMD as “software intended to be used for one or more medical purposes [that] performs these purposes without being part of a hardware medical device” (Health Canada 2019a). Medical purpose in this context is interpreted as “intended to acquire, process, or analyze a medical image, or information from an in vitro diagnostic device, or a measurement/signal from a monitoring device or imaging device” or “intended for the purpose of supporting or providing recommendations to health care professionals, patients or non-healthcare professional caregivers about prevention, treatment, or mitigation of a disease or condition” (Health Canada 2019a). The Canadian *Regulations* classify medical devices as either IVDD or non-IVDD. According to the International Medical Device Regulation (IMDRF), which Health Canada uses as a reference in certain instances (Health Canada 2019a) SaMD can be a medical device in itself and includes in vitro diagnostic devices (IVDD) (International Medical Device Regulators Forum (IMDRF) SaMD Working Group 2013). The distinction between the two is important, because the risk classification level for each differs,^1^ as will be examined more thoroughly in later parts of the paper. Notably, Health Canada’s guidance does not define the concept of software, which may leave room for uncertainty about what precisely the regulator’s oversight regime might target with respect to risk prediction (Health Canada 2019a). It is unclear, for example, whether regulators and developers should interpret the SaMD Guidelines as applying narrowly to individual risk prediction devices or more broadly to the underlying computational models. It is unclear whether this conceptual distinction is significant from the perspective of medical device regulation.

### Classification and intended use

Medical devices are bound by conditions that vary according to the degree of risk they pose to their users. The risk-based classification framework at the heart of Health Canada’s oversight regime consists of four tiers. Class I devices represent the lowest risk level and Class IV devices represent the highest (Medical Devices Regulations 1998). How a device is categorized is determined according to various factors, including a device’s degree of invasiveness, duration of contact, duration of use of the medical device, affected body system, and whether the device’s effects are local or systemic (Health Canada 2015). Decisions about how to classify a device according to this four-tier structure consider how the device manufacturer intends for their product to be used.^2^ This assessment permits the regulator to determine whether the product falls within the description of a medical device or whether special rules apply.^3^ The intended use of a medical device is a determining factor of its risk classification. As an example, an ECG machine intended to be used in primary care for routine check-ups, would usually be classified as a Class II device (Medical Devices Regulations 1998). The same device intended to be used in a critical care setting would be classified as a Class III device and be subject to stricter compliance rules (Medical Devices Regulations 1998). Because it is not directly relevant to our objective, we set aside the important distinction between invasive and non-invasive devices, which greatly affects how the MDD assesses risk (Medical Devices Regulations 1998). For the purposes of this essay, risk prediction models will generally not be invasive devices in the sense that they are not implanted or absorbed by the human body, though they may interact electronically with devices that do (Health Canada 2015).

How a device’s risk is assessed by the MDD directly impacts the regulatory requirements that must be met for the device to be marketed and sold in Canada. Recognizing their low risk level, Class I devices are only required to comply with general safety, effectiveness, and labeling requirements set out in the *Regulations* (Medical Devices Regulations 1998). Such devices are exempt from device licensing, quality management system certificate, and foreign manufacturers requirements (Medical Devices Regulations 1998). Premarket rules for Class II, III, and IV devices are more stringent, requiring applying or registering for a medical device license, in addition to compliance with safety, effectiveness, and labeling requirements. Licensing requirements likewise vary according to the risk class of a device (Medical Devices Regulations 1998). While the classification of a medical device is influenced by what the device’s manufacturer identifies as its intended use, the final determination lies with the MDD.

### SaMD regulation

With these background concepts set out, we now describe how SaMD is regulated in Canada. In the sub-parts above (1.1 and 1.2), we introduced key concepts in Canada’s regulatory regime and clarified that the MDD’s approach to regulating medical devices is fundamentally determined by the degree of risk medical devices pose to patients. In this sub-part we focus on how Health Canada applies this regime to SaMD. As a first step, Health Canada determines whether a software system meets the definition of SaMD. From there, the MDD determines whether SaMD should be excluded from regulation according to the Guidance. Finally, if not all the relevant exclusion criteria are met, the SaMD is categorized according to the level of risk it poses to patients.

As we noted above, Health Canada adopts the IMDRF’s definition of SaMD, according to which software can be considered a medical device if it (a) performs one or more medical purposes and (b) performs these purposes without being part of a hardware medical device (International Medical Device Regulators Forum (IMDRF) SaMD Working Group 2013; Health Canada 2019a). There are two important ways in which a software can be used for medical purposes. First, SaMD may provide the means and opportunity to capture, acquire, process, or analyze data from a hardware medical device to aid in the diagnosis or treatment of a patient. An example of this might be software that assesses medical images obtained from an MRI or other equipment. Second, SaMD may support or provide recommendations to healthcare professionals, patients, or non-healthcare professionals caregivers about preventing, diagnosing, treating, or mitigating a disease or condition (Health Canada 2011, 2019a). Health Canada excludes from the ambit of SaMD regulation software that does not have a direct impact on the diagnosis, treatment, or management of disease. Four general exclusion criteria limit the scope of SaMD oversight (Health Canada 2019a). These criteria are stated in the *SaMD Guidance Document* and effectively mean that software intended for administrative support of healthcare facilities, for clinical communication and workflow, for encouraging healthy lifestyles, or for maintaining electronic patient records are not subject to the rules surrounding medical devices. These exclusion criteria will be revisited later in point 2.2.

Once software is determined to be SaMD, and that the exclusion criteria above do not preclude the inclusion of the software in the regulatory regime, it must be classified based on its risk. The risk class is determined by the manufacturer and, in the case of Classes II–IV, confirmed by the Medical Devices Directorate when reviewing the medical device license application. Like for any medical device, manufacturers determine the risk class of their SaMD based on the intended use of their SaMD. This intended use is commonly included for instance in the intended use statement, labeling, instruction manuals, websites, or promotional material. Each SaMD will have its own risk classification, even if it interfaces with other SaMD, hardware medical devices, or functions as part of a medical system (Health Canada 2019a).

Both the IMDRF and Health Canada’s *SaMD Guidance Document* enunciate a two-factor criterion for determining the risk classification of SaMD.

First, the regulator reviews how significant software-generated information would be to a healthcare decision maker. SaMD can treat or diagnose a disease or condition, drive clinical management, or inform clinical management (Health Canada 2019a). Treating or diagnosing a disease or condition, for example, means that the information provided by the SaMD influences an immediate or near-term action to treat, prevent, or mitigate the condition by prescribing the use of other medical devices or medication. Diagnosis could alternatively refer to a process of screening for a disease or condition using sensors or data obtained from other medical devices (Health Canada 2019a). If the information or output provided by the software is used to advise on an immediate or near-term action in these terms, the software would be considered high risk (International Medical Device Regulators Forum (IMDRF) SaMD Working Group 2013; Health Canada 2019a). Driving clinical management refers to information provided by the SaMD that helps identify early signs of a disease or condition, thus helping to predict risks of such a disease or condition (Health Canada 2019a). If the information provided leads to clinical management by means of informing or aiding treatment or diagnosis, the software is deemed medium risk (Health Canada 2019a). Finally, informing clinical management means that the information provided by the SaMD does not trigger an immediate or near-term action. Instead, it merely provides information about treatments, diagnostics, prevention or mitigation measures or it aggregates relevant clinical information about diseases, conditions, drugs, medical devices or populations (Health Canada 2019a). Where the information provided by the software does not lead to immediate action and merely provides treatment or diagnosis options, or helps to aggregate relevant information, the software will be classified as low risk (Health Canada 2019a).

Second, in assessing SaMD’s risk classification, the regulator considers the state of the medical situation or condition. Such determination would be assessed using the following considerations: (a) critical conditions, which require accurate and/or timely immediate actions to avoid death, long-term disability or significant health deterioration or to mitigate impact to public health, (b) serious conditions, which require an accurate diagnosis or treatment to decrease the chances of long-term, irreversible health consequences, (c) non-serious conditions that benefit from timely diagnosis or treatments, but are not essential to alleviate the associated long-term irreversible health consequences (International Medical Device Regulators Forum (IMDRF) SaMD Working Group 2013; Thorogood et al. 2018; Health Canada 2019a). SaMD is considered to be used in critical conditions when the information it provides is vital to avoid a life-threatening or incurable state of health, when it leads to recommending needed major therapeutic interventions or actions that are time-critical, when the SaMD and the information it provides focus on a population that is fragile with respect to the disease or condition (e.g., pediatrics, high-risk populations) and when the SaMD and the information it outputs are intended for specialized trained users (Health Canada 2019a). SaMD is considered to be used in a serious situation or condition (a) when the information it provides is used to avoid unnecessary interventions (e.g., a biopsy) or to recommend a timely intervention to mitigate long-term irreversible consequences in conditions or diseases with a moderate progression and that are often curable, (b) when the needed intervention is not considered therapeutically major nor expected to be time-critical, (c) when the SaMD and the information it provides focus on a population that is not vulnerable with respect to that disease or condition, and (d) when the SaMD and the information it provides are intended for specialized trained or lay users (Health Canada 2019a). Finally, SaMD is deemed to be used in non-serious conditions when the information it outputs is associated with a disease or condition that has a slow predictable progression, that is effectively manageable, that only requires minor therapeutic interventions, or the SaMD and the information it outputs do not target patients and can be used by lay users (Health Canada 2019a).

In Table 1 below, we summarize how these factors generally influence the assignment of a risk category to SaMD. While this rubric captures how the MDD generally assesses risk, this determination is ultimately a decision of the regulatory body for which a degree of discretion is afforded.

**Table 1:** SaMD risk classification

State of healthcare situation of condition	Significance of information provided by SaMD to healthcare decision		
	Useful for treatment or diagnosis	Drives clinical management	Informs clinical management
Critical	III	III	I or II
Serious	II or III*	II or III*	I or II
Non-serious	I or II	I or II	I or II

Adapted from (Health Canada 2019a) at 14

*In the case where an erroneous result could lead to immediate danger, the SaMD would be classified as risk class III, instead of II

To provide a more concrete idea of how SaMD will be classified, both the IMDRF and Health Canada provide examples of SaMD risk categorization. The examples suggest that (a) software that uses data from individuals for predicting risk scores in healthy populations for risk of migraines (International Medical Device Regulators Forum (IMDRF) SaMD Working Group 2013) and software intended to receive alerts from a patient’s hospital monitor without providing physiological data, measurement or medical images (Health Canada 2011) would be considered Class I devices: (b) software that uses data from individuals for predicting risk scores for developing a stroke or heart disease in order to create prevention or interventional strategies (International Medical Device Regulators Forum (IMDRF) SaMD Working Group 2013) and software that calculates percent breast density from the same digital mammograms that radiologists view in breast screening exams to aid in the assessment of breast tissue composition with respect to determining the risk of developing cancer (Health Canada 2011) would be considered Class II devices, (c) software intended to tailor radiation treatment dosage using patient information that provides specific parameters tailored for a particular tumor and patient (International Medical Device Regulators Forum (IMDRF) SaMD Working Group 2013) and software that performs diagnostic image analysis for making treatment decisions regarding patients with acute strokes (Health Canada 2011) would be Class III devices, and (d) software that uses data to predict risk scores in high-risk populations for developing preventive or intervention strategies for colorectal cancer would also be categorized as Class III (International Medical Device Regulators Forum (IMDRF) SaMD Working Group 2013; Thorogood et al. 2018).

Finally, although SaMD may run on different operating systems and virtual environments, they must nevertheless be designed to be safe and perform as intended, just as any other medical device. This means that manufacturers must ensure the software’s repeatability, reliability, and compatibility with respect to its use with any specific hardware or platform, IT networks, and security measures (Medical Devices Regulations 1998).

As we mentioned above, SaMD can sometimes be categorized as IVDD (International Medical Device Regulators Forum (IMDRF) SaMD Working Group 2013; Health Canada 2019a). Though a software’s IVDD character is an important factor in considering risk classification and other applicable rules, we do not directly address these concerns here because, though SaMD may qualify as an IVDD, this is not likely the case for risk prediction models.

## Legal framework applicable to risk prediction models in Canada

In this part, we give a short overview of cancer risk prediction models and describe how, following the framework in the part above, they are likely to be regulated in Canada. Risk prediction models use risk factor data such as health, family history, lifestyle, genetic data, PRS, sex, gender, age, race/ethnicity and ancestry for the purpose of estimating the probability that an individual will develop a particular health outcome (Grant et al. 2018; Barda et al. 2020). These predictions are intended to inform, influence, or suggest to a physician to recommend that a patient undergo specific screening or diagnosis strategies that could lead to a more effective and timely treatment or surgical intervention. In some cases, these predictions may be used in the design of personalized treatments (Grant et al. 2018). This means that risk prediction models may have a medical purpose, and as a result, be captured in the definition of SaMD we described above. However, for the risk prediction model to be considered a medical device, it must also be established that the risk prediction model (software) does not fall in the exclusion criteria.

Algorithms implemented in risk prediction models typically draw on a combination of disease characteristics and personal health information to produce insights into the risk levels of individuals. These models are becoming more complex, increasingly integrating genetic screening data and polygenic risk scores as well as calculating risk for multiple types of a disease. Indeed, some software for example, can now implement multiple models and therefore cover multiple cancer types (Wang et al. 2014). Early data show that adding genetic risk factors improves refinement and cancer prediction, increasing the clinical potential of these tools. Overall, the sensitivity and specificity of risk prediction tools vary however, and their clinical utility and optimal implementation is still being established on a case-by-case basis (Usher-Smith et al. 2015).

### An example: the CanRisk tool using the BOADICEA algorithm

An example of a cancer risk prediction model may be helpful for better contextualizing the regulatory regime to which these tools are subject. The Breast and Ovarian Analysis of Disease Incidence and Carrier Estimation Algorithm (BOADICEA) is a statistical model that processes genetic and other health data to estimate an individual’s future risk level of developing breast and ovarian cancer (Lee et al. 2014, 2019). Output risk data generated by the model could be used to inform and drive physicians to recommend early prevention measures to certain potential breast or ovarian cancer patients. Though the BOADICEA algorithm was programmed to capture incidences of breast and ovarian cancer in the British population, its scope of use has since been extended to assess incidences in Australia, Canada, Denmark, Finland, Iceland, New Zealand, Norway, Sweden, and the United States (Lee et al. 2014). It is regulated in the European Union (EU) and qualifies as a Software as a Medical Device (SaMD) as per their medical devices regulatory framework (namely the EU Medical Device Regulation (Regulation (EU) 2017/745 of the European Parliament 2017) and the European Commission Guidelines on medical devices (European Commission 2015)). To be made available in the EU, the BOADICEA risk model had to receive a Conformité Européenne (CE) marking in the EU economic market. This process is generally long, costly and time-consuming. This approach differs from the United States, where SaMD that are intended to inform clinical management would not meet the criteria for regulation if they are intended for a health care professional to independently review and understand (Artificial intelligence in EU medical device legislation 2020). The EU regulatory framework has therefore a larger scope than that of the US, incorporating more software intended to inform clinical management under its remit. That being said, the US has recently completed its Pre-Cert pilot program (September 2022), which aimed to explore whether certain methods for evaluating safety and effectiveness throughout the product life cycle could be used to assess medical device software safety and effectiveness (The software precertification (Pre-Cert) pilot program: Tailored total product lifecycle approaches and key findings 2022). It is foreseen that, in the future, similar evaluations as those performed by the EU will be implemented in the US, with shortened protocols.

Initially implemented as a stand-alone program, BOADICEA has been configured as a web-accessible application, through the CanRisk web tool, to better suit clinical use and to encourage more widespread adoption (The software precertification (Pre-Cert) pilot program 2022).^4^ This tool enables healthcare providers to run risk calculations directly within their own clinical software and existing electronic health record systems (Lee et al. 2014). Newer, updated versions continue to improve the tool to include additional relevant mutations and variants, and lifestyle/hormonal risk factors (e.g., parity, age at first live birth, age at menarche, age at menopause, oral contraceptive use, menopause hormone therapy, body mass index, and alcohol intake) (Lee et al. 2019). For greater clarity, we refer in our discussion here primarily to the CanRisk tool that includes the BOADICEA algorithm.

### Applying the regulatory framework in Canada

As we described above, Health Canada decides whether and how to regulate SaMD according to a three-step process. First, the regulator determines whether the software broadly meets the definition of SaMD set out in the guidance (Health Canada 2019a). CanRisk uses genetic and other health data to estimate future risk level of developing cancer, which would likely be deemed to align with the broad definition of SaMD. Aside from serving as a source of data to aid in the prediction and potential diagnosis or treatment of a patient, a risk prediction model could inform and drive physicians to recommend early prevention measures to certain potential cancer patients. This appears in agreement with the general requirements of medical purpose set out in Canada’s definition and interpretation of medical devices and medical purposes, namely, that the device is intended to support or provide recommendations about the diagnosis, mitigation, and treatment of a disease (Food and Drugs Act 1985; Health Canada 2019a; Medical devices regulations 1998).

Second, Health Canada determines whether the software under review is excluded from the oversight regime. The *SaMD Guidance* sets out four exclusion criteria and a device that meets all of them will not be regulated. But knowing whether these factors are met in the case of risk prediction models is difficult. For one thing, the interpretation and understanding of the purpose and functions of the risk prediction model as envisioned and described by the manufacturer, which can also vary and evolve, will greatly influence the interpretation of whether the exclusion criteria are met (Health Canada 2019a). Furthermore, and perhaps more pressingly, CanRisk provides information that, though potentially clinically useful, may only influence patient care indirectly, sometimes many years before a therapeutic or preventive intervention may be warranted. These factors complicate reaching a straight, definitive conclusion as to the likely exclusion of risk prediction models from regulatory oversight. Health Canada’s exclusion criteria are unclear, even without these complicating factors. In Table 2 below, we outline the exclusion criteria as they are listed in the *SaMD Guidance* Document, as a way of illustrating the difficulty of consistently assessing the appropriate category of risk prediction models in this regime.

**Table 2:** Exclusion criteria as stated in the Health Canada SaMD Guidance Document

Exclusion criteria	Interpretation adapted from the document
1. Software not intended to acquire, process or analyze a medical image or information from an in vitro or monitoring device	Software that acquires data or information from medical devices for the sole purpose of display, storage, transfer or format conversion
2. Software intended to display, analyze or print medical information	Software that matches medical information to reference information, including patient symptoms with best treatment guidelines for common illnesses or to identify possible drug interactions
3. Software intended only to support a healthcare professional, patient or non-healthcare professional caregiver in making decisions about prevention, diagnosis or treatment of a disease or condition	This software is intended to inform clinical/patient management and it is excluded because merely informing infers that the information provided will not trigger an immediate or near-term action Software used to treat, diagnose or drive clinical management does not usually fall under this criterion, as treatment or diagnoses infers that the information provided will be used to take an immediate or near-term action
4. Software not intended to replace the clinical judgement of a healthcare professional to make a clinical diagnosis or treatment decision regarding a patient	The intended user is able to reach a recommendation independently without relying primarily on the software, such as software that provides a convenient way to preform simple medical calculations

Adapted from (Health Canada 2019a) at 9–10

Applying each of these criteria in turn, the CanRisk tool and other risk prediction models will likely easily meet criteria (1) and (2). Risk prediction models do not typically acquire, process, or analyze medical images or information from in vitro or monitoring devices. Though information processed by risk prediction models may originate from several sources, it will not generally collect data obtained from in vitro or monitoring devices, but rather from published data and stand-alone testing and imagining. Risk prediction models will likewise tend to meet criteria (2) insofar as they analyze medical information.

On the question of criteria (3), risk prediction models are usually intended only to play a supportive role in medical care and will not directly provide diagnoses. Criteria 3 then is most likely satisfied as well. As for Criteria 4, in its interpretative guide to criteria, Health Canada suggests that software not intended to replace the clinical judgement of a healthcare professional will generally only provide recommendations that can be reached independently by the software’s intended user, without their relying on the software in question. This is where uncertainty in interpretation may arise. If the risk prediction model’s output does not replace the clinical judgement of a healthcare professional, then it could be argued that it also satisfies point 4 and is therefore excluded from the regulatory process. That said, it could also be argued that risk prediction, insofar as it considers numerous interrelated biological and clinical factors to provide accurate and more precise defined risk assessments related to specific conditions and treatments, is doing something that cannot be easily replicated by a clinician without the tool’s assistance. This argument could be especially persuasive in the context of constantly updated versions that incorporate new risk factors from multiple sources that would be unfeasible to assess by a healthcare professional on their own. The key, then, according to Health Canada’s guidance, will be whether the clinician is still able to independently review the basis for the recommendations presented by the software. If the answer is yes, then the risk prediction model would meet criteria 4. As will be seen in the stakeholders’ perspectives section below (Stakeholder Perspectives), a recommendation is made on the importance of risk control both at the level of the software and at the time of its clinical use. Such risk control seems to imply that an ability to independently review the basis for the recommendation presented by the software is feasible.

To be sure, risk prediction models as we have described them do not aim to *replace* the clinical judgment of healthcare professionals. In fact, although some are available publicly, they are only meant to be used by healthcare professionals and researchers, as is the case with CanRisk.^5^ Health Canada’s Classification Examples document, which is referenced in its Guidance (Health Canada 2019a), helps to contextualize this argument. In the case of a hypothetical prediction model for diabetes presented in the *SaMD Classification Examples* Document, whether to exclude the software from regulation comes down to a question of whether the model provides “a diagnostic output that the healthcare provider would otherwise not have access to,” (Health Canada 2011) Insofar as a model provides diagnostic outputs to which a healthcare provider would otherwise not have access, Health Canada suggests, it fails to meet exclusion criteria (4) above and would be subject to regulation. However, the essential function undertaken by risk prediction models such as the CanRisk tool is to assist clinicians in making medical decisions about the management of future disease risk: a medical purpose that is made more efficient and precise by using a risk prediction model. This is supported by stakeholders (Stakeholder Perspectives) who see the output of such software as not being individualized but stratified by risk group; just one of several elements to consider in clinical practice regarding a medical condition that is not yet present in an individual patient.

At present, it is uncertain exactly how Health Canada will weigh these exclusion criteria. This is a point raised consistently in the stakeholder interviews presented below. The SaMD regulatory regime is new and a precise mapping of the way its rules will be applied has not yet been compellingly set out. We suggest that based on Health Canada’s Guidance on SaMD and supplementary Examples document, that a reasonable argument could be made that risk prediction models such as CanRisk should satisfy each of the exclusion criteria described and therefore be excluded from regulatory oversight. This being the case, the analysis would end here. This is, in our view, the most likely scenario with respect to CanRisk. Conversely, should Health Canada hypothetically determine that one or more exclusion criteria are *not* met, then the analysis would proceed to the third and final step: risk classification. In our view, it is likely that risk prediction models that perform functions like those described above would then be considered as falling under Class II SaMD. This opinion is founded on two broad reasons. First, the outcomes (information or results) provided by these kinds of models could be seen as having the effect of primarily driving clinical/patient management (Health Canada 2019a; Lee et al. 2019). Second, risk prediction models would generally not directly diagnose disease or solely determine an appropriate treatment or intervention (Lee et al. 2019). Both of these considerations are consistent with a Class II risk determination. This view is also supported in the Classification Examples document. There, it is indicated that a Class II determination is appropriate if the decisions given by a device would not lead to immediate danger for the patient (Health Canada 2011).

### The regulatory approval process in Canada

As mentioned above, should Health Canada hypothetically determine that one or more exclusion criteria are not met, the regulatory approval process for a class II SaMD would require the submission of a *Medical device license application and fee form*, the submission of an *Attestation of objective evidence for safety and effectiveness*, a *QMS certificate*, and a Health Canada compliant label (Medical Devices Regulations 1998). Additionally, regardless of the risk class assigned, to comply with the *Regulations*, the manufacturers of a risk prediction model must ensure that the model performs as intended,^6^ is reliable (i.e., that is maintained so it performs as intended by the manufacturer) and compatible with the hardware, operating systems, virtual environments and platforms it is intended to be used with (Medical Devices Regulations 1998; Health Canada 2011, 2019c), including Canadian healthcare platforms and health records systems. Manufacturers will also need to identify any inherent risk(s) and eliminate or reduce such risk(s) in conformity with the safety requirements. The risk prediction model must also function as intended without deteriorating under normal use to such a degree that its safety be affected (Medical Devices Regulations 1998). In addition, the labeling requirements need to be followed by including all pertinent information regarding the device on the device label.^7^ Each of the risk prediction model’s manufacturers, importers, and distributors must also maintain a distribution record of each of the devices under their purview, as well as records of any problems associated with the device’s performance and actions taken to address those problems, unless they are retailers or a healthcare facility in respect of devices distributed for use within that facility (Medical devices regulations 1998). These factors are outlined in Table 3 below, for all device classes.

**Table 3:**
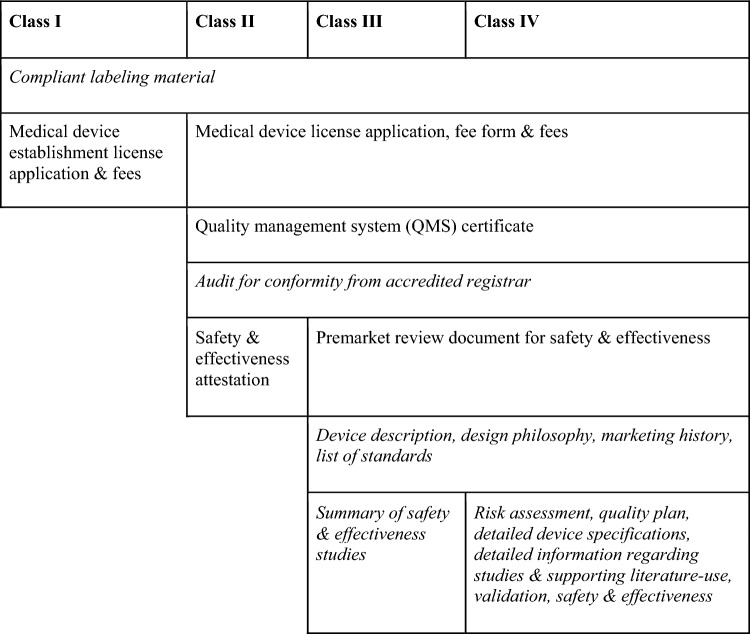
Application requirements by risk class

Adapted from (Medical Devices Regulations 1998)

## Stakeholder perspectives

Recognizing the inherent uncertainties of the Canadian regulatory framework, we sought out the perspectives of expert stakeholders active in this area. Between November 2019 and August 2021, our research team conducted semi-structured interviews with members of key stakeholder groups (*n = *10 participants in total): researchers and developers (*n = *3), regulators and decision-makers (*n = *4), and advocates for the medical technology industry (*n = *2) as well as from a patient organization (*n = *1). Members of these groups we interviewed all had a close interest in the evolution of the new regulatory process. Their perspectives are relevant to practical aspects, implications, and concerns regarding the potential regulation of susceptive cancer risk prediction software. This includes identifying areas that may need clarification and issues that may need to be addressed for the effective and efficient implementation of cancer risk prediction models.

Ethical approval for the exploratory interview portion of the study was obtained from the Institutional Review Board of McGill University’s Faculty of Medicine (#A03-B14-19A). Purposive sampling was used to recruit potential participants via information found on publicly available websites and the authors’ professional networks. In addition to recruiting from Canada, participants were also recruited from France and the UK, as international perspectives were deemed important to the discussion. Interviews were audio-recorded and lasted between 45 min and an hour and a half. Participants provided written consent. Questions were tailored to each stakeholder’s group category and location. Notably, interviews were tied to the larger grant associated with this study and extended beyond this paper in focus and scope.^8^ However, they all addressed two topics directly relevant to this paper, allowing for systemic analysis of transcripts for related data. Those topics were: (1) perspectives regarding risks and benefits of cancer risk prediction models and (2) practical questions regarding the regulation of medical devices in Canada. Due to the exploratory focus of the paper and the nature of the data,^9^ directed content analysis was used to represent and summarize themes and core ideas from the interviews relevant to our research question (Drisko and Maschi 2015). T.K. performed the content analysis which was then independently validated by a research assistant. The software Excel was used to manage emergent themes relevant to the two subtopics by stakeholder group. As is the nature of qualitative data, themes raised are topical perspectives from this collection of stakeholders and not meant to be interpreted as broadly representative or comprehensive; rather they provide valuable early insight regarding the kinds of issues that may need to be addressed as prescriptive cancer risk prediction models gain traction and face regulation for clinical use. Methods and findings are reported in light of COREQ, a checklist that promotes rigor during qualitative analysis (Tong et al. 2007).

### Risks and benefits of cancer risk prediction

As governance of SaMD is approached through a risk-based model, and software predicting risk of future cancer incidence are novel emerging products, stakeholders’ views on the risks and benefits of their clinical implementation were of interest. Table 4 summarizes risks and benefits brought forward by stakeholders during interviews.

**Table 4:**
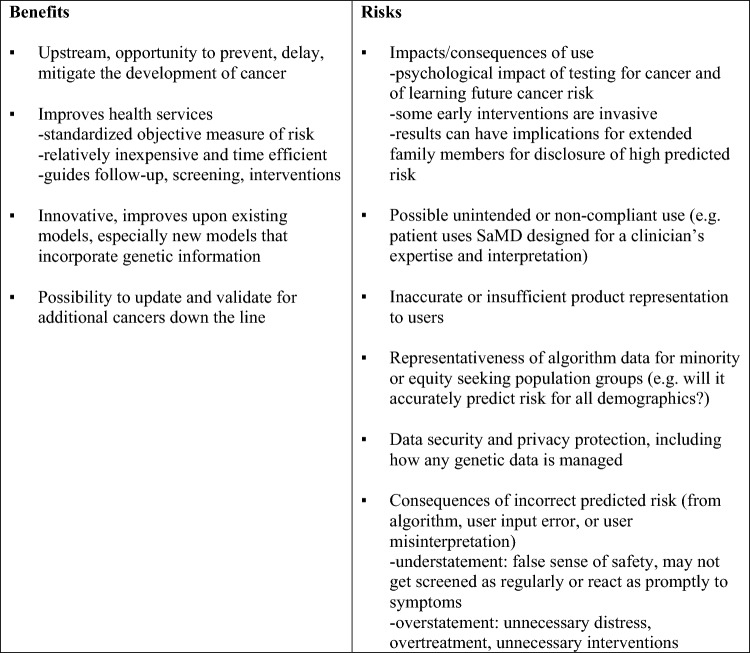
Stakeholder perspectives on risks and benefits of cancer risk prediction models

In parallel to the double-factor criterion for determining risk classification in the SaMD guidance document (SaMD Regulation), stakeholders brought up that the specific medical condition at stake and the specific role of a given risk prediction model in the diagnosis odyssey and clinical workflow are important to overall risk. They emphasized that because these models concern cancer, predicted risk brings serious implications and consequences. For instance, a model researcher/developer spoke to an ‘enormous’ responsibility felt while developing code, knowing it will be part of serious medical decision-making, and appreciating regulatory processes for helping to make such software safer. Stakeholders predicted the models would fall somewhere between informing and driving clinical management and added that the actual significance of the software-generated information to clinicians would be closely tied to product claims, ease of use, and how well a given tool predicts risk. Interestingly, several stakeholders believed that susceptive cancer risk prediction models pose a higher risk than those for already symptomatic cancer, as they tell patients about their futures and set off a string of consequences that would not have occurred otherwise. Further, as one researcher pointed out, this string of consequences occurs even though being in the highest cancer risk category as an individual does not indicate a certainty of getting cancer. Taking the example of the CanRisk Tool, the researcher contended that receiving a high-risk designation could have a significant emotional impact on the patient as well as potentially lead to intensive medical follow-up, informing and testing biological family members, and a prophylactic mastectomy. For these reasons, stakeholders discussed how risk prediction tools require two types of risk controls: 1) for the software itself and 2) for how it is used clinically, including any unintended or unanticipated consequences once the software has been approved and is in clinical use. At the same time, they emphasized that advanced warning on the development of cancer could be of high benefit to society and that there are both significant risks and significant benefits to consider.

Notably, some stakeholders were unclear about whether risk prediction tools would qualify as medical devices in Canada (Applying the Regulatory Framework in Canada). This was mainly due to needing device specific information-particularly about intended use. Other reasons included that the output of such software is not individualized but stratified by risk group, that the output is just one piece of information in clinical management, and that the medical condition is not yet present. When asked to anticipate potential risk classification should risk prediction models be subject to regulation, stakeholders gave a range of responses. The advocates, researchers and developers predicted relatively lower risk classifications (1 or 2, with 4 stakeholders predicting it could be level 1). The regulators and decision-makers stated that they would require detailed information specific to a particular tool to provide an actual estimate—reiterating the importance of patient safety and product effectiveness, labeling and usability. They postulated such models could be low to medium risk (2 or 3), depending on their specific characteristics.

### Practical considerations regarding the regulatory approval process

The perspectives and concerns of stakeholders are also valuable in elucidating the practical implications and unclear aspects of the SaMD regulatory approval process for cancer risk prediction tools (The Regulatory Approval Process in Canada). Two themes emerged from the interviews: the need to clarify and streamline the approval process for SaMD and for international harmonization.

Stakeholders were in general agreement about the need to continue to update the Canadian regulatory framework to facilitate the classification and approval of emerging technologies. Regulators and decision-makers noted Health Canada’s established priority to modernize regulations as they are aware there are gaps in how the system will address novel devices. They pointed to the SaMD guidance document as a first move towards formalization and clarification and encouraged engagement with regulatory requirements and bodies early in software development to facilitate the process for applicants. Advocates, researchers and developers asked for further clarity on regulation for cancer risk prediction models and advocated for a simpler, more streamlined process with a more efficient timeline to market/patient access. As one medical industry advocate put it, innovative technologies can get ‘stuck’ in the approval process. The patient advocate asked for more public transparency about the approval process, including access to information about submissions from developers. Stakeholders affiliated with the cancer risk prediction industry described risk classification and application requirements as uncertain, technical and involved and asserted that this costs extraneous resources, labor, and time. One researcher spoke to the ‘cultural gap’ between research and development activities and expertise in regulatory compliance, citing the need to hire private specialists to prepare regulatory applications and that even then, consultants can give conflicting interpretations. The researcher shared the anecdote of an application in another jurisdiction that took a year and a half to prepare, during which they had four staff members dedicated to the regulatory process and only two working on the SaMD itself. A medical industry advocate observed that the most clinically beneficial or important products can be the hardest to get to market as they bring the highest costs and compliance requirements (see Table 3) and that this can disincentivize critical innovation. There were also concerns about pursuing the wrong regulatory path and wasting time and resources and about updates and improvements in cancer risk prediction tools that would require new regulatory applications.

A second significant theme for stakeholders was the importance of international harmonization in regulatory frameworks for medical devices. Currently, developers have to prepare separate applications for each jurisdiction. Stakeholders from the risk prediction model industry saw this as an important barrier to seeking regulatory approval in Canada. One medical technology industry advocate worried that disparate regulatory requirements and approaches across jurisdictions might be hurting innovation and international business interest, citing Canada’s requirements as relatively stringent and Canada’s share of the global market as relatively small. Similarly, a researcher stated that a potential consequence of regulation being perceived as overly burdensome is ‘medical tourism’ on the part of companies seeking out easy markets or skirting local regulation to market unauthorized products. A second medical technology industry advocate asserted that international consistency and harmonization across jurisdictions is increasingly important as there are more global projects and products and it facilitates cooperation when all parties have a similar regulatory culture. The patient organization advocate expressed that increased harmonization would reduce equity issues across jurisdictions in terms of access to medical devices. Finally, the regulators and decision-makers agreed about the need for greater harmonization and discussed international initiatives already underway to align regulatory policy approaches and better align submission requirements.

## Conclusion

Emerging cancer risk prediction models raise new questions about the regulation of medical devices. It is not yet clear how these models will be regulated, and the available guidance remains insufficiently detailed. This paper aims to address this novel regulatory question by presenting an initial assessment of the legal status likely applicable to risk prediction models such as the CanRisk tool. While it focuses on the Canadian regulatory framework it also identifies some aspects of European and U.S. regulations in this domain. We have demonstrated that the Canadian process is stringent, also giving rise to potentially contradictory interpretations and indicating that the regulator is likely to assess these new technologies on a context-dependent and case-by-case basis. This, as we have endeavored to show, gives rise to uncertainty. We engaged with stakeholders to better understand how the legal framework for cancer risk prediction models impacts their implementation and further development. These stakeholders generally highlight the need for greater clarity around SaMD and demonstrate how regulation perceived as convoluted or overly burdensome can discourage innovation and compliance. Yet, looking at the current evolution of medical devices regulations in Canada, the U.S. and Europe, following a period of observation, governments and agencies are now seemingly moving towards the imposition of more stringent regulatory oversight of risk prediction models. More rigid oversight may impede research and improvement of these models by the academic community but it does send a clear signal to private companies that the field of risk prediction models has arrived at a level of maturity that may warrant their interest.

We aim with this contribution to initiate discussion about a more optimal legal framework for risk prediction models in Canada. This discussion is becoming increasingly relevant because, as our stakeholder participants underline, uncertain or excessively burdensome regulatory frameworks strongly impact the implementation of novel medical technologies. All of this ultimately makes it more difficult for the Canadian public to access new and potentially lifesaving approaches to medical care. As health systems around the world move toward integrating predictive and precision medicine in oncology, it is essential that policymakers prioritize predictability, international coherence and scientific rigor in its prediction model regulation. The potential of risk prediction models to reduce the impacts of cancer in Canadian society alone is testament to the urgent need to clarify and update our SaMD regulatory framework.

## Data Availability

Data sharing not applicable to this article as participants did not provide consent for the sharing of interview transcripts with parties other than the researchers.

## Abbreviations

SaMD: Software as a medical device
MDD: Medical Devices Directorate of Health Canada
DHRD: Digital Health Review Division, a division of the MDD

## Footnotes

1 The risk classification for non-IVDD’s is based on the level of invasiveness, tissue/organ exposure, potential for infection, and energy exposure. The risk classification of IVDD’s, on the other hand, is mostly based on clinical application (screening, diagnosis, monitoring, prognosis, and predisposition), expertise of intended user, the important of the information to the diagnosis, the nature of the disease, and the impact of the diagnostic test results to the individual concerned (Health Canada 2018b).

2 The intended use of the device is evidenced by the device’s label, directions, brochures and operating manuals.

3 These special rules regulate devices intended to be used for disinfecting or sterilizing blood, tissues or organs intended for transfusion or transplantation, for disinfecting or sterilizing a medical device, devices manufactured from or that incorporate human or animal cells or tissues or their derivatives or that are manufactured from or incorporate a product produced through the use of recombinant DNA technology, or devices that are intended to come into contact with intact skin, devices that are intended to be sold to a health professional or dispenser for the specific purpose of configuration or arrangement into a mold or shape to meet the needs of an individual, as well as breast implants and tissue expanders for breast reconstruction and augmentation (Health Canada 2015).

4 At a basic level, web services and web applications are very similar. They both operate over https. However, a web service is a technology by which two or more remote web applications interact with each other over the network or the Internet. It is essentially a collection of open protocols and standards used for exchanging data between applications or systems allowing programs to communicate and collaborate with each other, without necessarily connecting human beings as clients. A web application on the other hand, is a technology used to perform a function and to use some of the web applications’ features in ways that may enable interactions with human beings as clients. See (Ashwini 2016); Web service vs web application 2016).

5 (CanRisk FAQs: Who can use the CanRisk tool? n.d.) “The CanRisk Tool has been approved for use by healthcare professionals and researchers in the UK and European Economic Area. For legal reasons, we regret that it cannot be used by members of the public.”.

6 That is, it must provide a risk-based screening possibility that provides personalized risk assessment for early detection of high-risk women.

7 Such information includes the name of the device, the name and address of the manufacturer, uses for which BOADICEA was manufactured, as well as the performance specifications and the directions for use, all of which must be at least in English or French, visible under normal circumstances, and placed on the outside of the package. (Medical devices regulations 1998) ss 21–23.

8 PERSPECTIVE I&I project (2018-present), *see* Acknowledgment section.

9 The relatively small number of participants and requests for anonymity (to be identified by stakeholder group only and to be paraphrased rather than directly quoted).
